# Two-Dimensional Metal-Organic Framework Incorporated Highly Polar PVDF for Dielectric Energy Storage and Mechanical Energy Harvesting

**DOI:** 10.3390/nano13061098

**Published:** 2023-03-19

**Authors:** Abhishek Sasmal, Jaganathan Senthilnathan, Arunachalakasi Arockiarajan, Masahiro Yoshimura

**Affiliations:** 1 Department of Applied Mechanics, Indian Institute of Technology Madras (IIT Madras), Chennai 600036, Tamil Nadu, India; am22r001@smail.iitm.ac.in; 2 Department of Civil Engineering, Indian Institute of Technology Madras (IIT Madras), Chennai 600036, Tamil Nadu, India; 3 Centre of Excellence in Ceramics Technologies for Futuristic Mobility, Indian Institute of Technology Madras (IIT Madras), Chennai 600036, Tamil Nadu, India; 4 Department of Materials Science and Engineering, National Cheng Kung University, Tainan 70101, Taiwan

**Keywords:** metal-organic framework, PVDF, polar phase, dielectric energy storage, piezoelectric nanogenerator, triboelectric nanogenerator

## Abstract

Here, we introduce a 2D metal-organic framework (MOF) into the poly(vinylidene fluoride) (PVDF) matrix, which has been comparatively less explored in this field. Highly 2D Ni-MOF has been synthesized in this regard via hydrothermal route and has been incorporated into PVDF matrix via solvent casting technique with ultralow filler (0.5 wt%) loading. The polar phase percentage of 0.5 wt% Ni-MOF loaded PVDF film (NPVDF) has been found to be increased to ~85% from a value of ~55% for neat PVDF. The ultralow filler loading has inhibited the easy breakdown path along with increased dielectric permittivity and hence has enhanced the energy storage performance. On the other hand, significantly enriched polarity and Young’s Modulus has helped in improving its mechanical energy harvesting performance, thereby enhancing the human motion interactive sensing activities. The piezoelectric and piezo-tribo hybrid devices made up of NPVDF film have shown improved output power density of ~3.26 and 31 μW/cm^2^ compared to those of the piezoelectric and piezo-tribo hybrid devices comprising of neat PVDF (output power density ~0.6 and 17 μW/cm^2^, respectively). The developed composite can thus be considered an excellent candidate for multifunctional applications.

## 1. Introduction

Polymers, hydrogels, biomolecules, and their nanocomposites are perhaps the most widely studied materials class in present days, which show a wide variety of practical applications, including dielectric energy storage, electrode materials for supercapacitors, mechanical energy harvesting, separation membranes, and many others [1,2,3,4,5,6,7,8]. In this regard, poly(vinylidene fluoride) (PVDF) is perhaps the most widely studied material in recent times due to its cost-effectiveness, easy synthesis procedure, and several other unique and suitable functional properties [9,10]. The electroactive performances of PVDF greatly depend on its polarity. In normal conditions, PVDF always tries to exhibit the non-polar α phase due to its energetically most favorable TG^+^TG^−^ chain conformation [11]. But, for good output electroactive performances, polar β and/or γ phases with TTTT and T_3_G^+^T_3_G^−^ chain conformations, respectively, are necessary. The amount of β phase (exhibiting the highest dipole moment) in PVDF-based composite films strongly impacts their output electroactive properties such as dielectric permittivity and piezoelectric activity [11]. There have been tremendous efforts to improve the polarity of PVDF by following a variety of unique techniques [12]. Incorporation of nanomaterials into a PVDF matrix is such a technique that is easy and cost-effective and hence has been explored most widely [11,12]. The basic idea of incorporating nanomaterials into the PVDF matrix is to induce strong interfacial interaction between PVDF dipoles (–CH_2_ and –CF_2_ dipoles) and the surface of the incorporated nanomaterials so that the TG^+^TG^−^ chain conformation of α phase transforms to TTTT and/or T_3_G^+^T_3_G^−^ chain conformations of β and γ phases, respectively, through the dipole alignment caused by this interaction [11,12,13]. Moreover, different functional properties of nanomaterials can be incorporated into PVDF-based composites for the corresponding performance of the final device, along with the applications related to polarity. Inorganic oxides, due to their various physiochemical advantages and unique electronic and functional properties [14,15,16,17,18,19,20], can be easily used as fillers in PVDF and hence under tremendous research attention in this regard [14,19].

Different techniques like surface modification of fillers [21], formation of core-shell fillers [22], etc., have been explicitly explored in this regard in order to strengthen this interfacial interaction and hence improve the polarity of PVDF-based composites [21,22,23]. These surface or interface modification techniques require difficult chemical routes. Therefore, researchers are now focused on fillers exhibiting a high aspect ratio which can render highly active sites for interfacial interaction [24,25]. Among the different high aspect ratio fillers, two-dimensional (2D) materials have attracted much attention in the present day. Owing to their high surface area, they can render a high number of active sites for interfacial interaction which can improve the polarity of the resulting PVDF-based composites [26,27]. Moreover, due to their excellent electronic and optoelectronic properties, the development and usability of 2D nanomaterials have attained immense attraction in recent times to researchers all over the world for several real-life applications. These functional properties of 2D materials can further be combined with the output electroactive properties of their PVDF-based composites [28]. However, this article is only focused on the electroactive performance tuning of PVDF caused by 2D nanomaterials incorporation into its matrix.

In this regard, different 2D materials such as hexagonal boron nitride (hBN) [29], MoS_2_ [27], WS_2_ [30], MXene [31], g-C_3_N_4_ [32], graphite platelet [33], reduced graphene oxide (rGO) [34], and many others have been explored as filler to PVDF matrix in order to improve the polarity, mechanical energy harvesting activity, energy storage performance and many other functional properties of the resulting composite systems. 2D Metal-organic frameworks (MOFs) are recently gaining potential attention in this field. They have also been applied in other polymer matrices for piezoelectric energy harvesting applications [35]. However, MOFs, either in 3D or in 2D morphology, have been comparatively less explored as fillers in PVDF matrices for different real-life applications [36,37,38,39]. They are a very emerging class of materials in present days for their environmental, catalytic, sensing, and biomedical applications and usability in the field of renewable energy [36]. These properties can further be coupled with the electroactive performances of PVDF, which is out of the scope of the present work. Here we mainly focus on investigating the energy storage and mechanical energy harvesting performance of PVDF tuned by 2D MOF nanofiller addition. Owing to their porous structure and organic bonds, MOFs possess better compatibility with the PVDF matrix compared to that inorganic fillers [36,40]. Moreover, when the metal site of MOF is selected as the transition metals such as Mn, Fe, Co, Ni, etc., spontaneous polarization occurs between organic bonds and metal sites, which in turn forms unique nanoelectric domains, thereby improving the ferroelectric properties [40,41]. Here in the present work, we, therefore, synthesize less explored 2D MOF using Ni as the metal site (MIL-53 (Ni)) and incorporate it into the PVDF matrix.

Most often, it has been observed that the improvement in energy storage density of PVDF caused by filler addition is accompanied by a reduction in storage efficiency which is attributed to the formation of conducting pathways, formation of defects, and non-uniformity in electric field distribution within the composite films due to higher amount of filler loading [42]. We, therefore, focus here on fabricating PVDF-based composite films by very low-content Ni-MOF loading. Owing to their 2D morphology, the synthesized Ni-MOF is expected to significantly improve the polarity of PVDF even for low concentrations of filler loading, which can improve its mechanical energy harvesting activity. On the other hand, due to its better compatibility with the PVDF matrix and low concentration loading, the electric field distribution is expected to be uniform within the composite films, which in turn may improve their breakdown strength and hence the energy storage performance [43]. Considering all these advantages, low content (0.5 wt%) hydrothermally synthesized 2D Ni-MOF has been incorporated here into the PVDF matrix, and its effect on the polarity, electroactive properties, energy storage performance and mechanical energy harvesting activity of the resulting composite system has been explicitly investigated here. The applicability of the developed mechanical energy harvesters for low-power electronics and self-powered sensing has also been demonstrated here.

## 2. Materials and Methods

### 2.1. Synthesis of 2D Ni-MOF

The MIL-53 (Ni) nanosheets were synthesized via hydrothermal technique. Typically, 2.88 mM (0.4784 g) of terephthalic acid (Benzene-1,4-dicarboxylic acid (BDC)) (SRL Chemical, 98%) was first dissolved in a mixed solvent of 70 mL Dimethylformamide (DMF) (Merck, Mumbai, India, 5 mL deionized water (DI water) (Evergreen, Chennai, India) and 5 mL absolute ethanol (Merck) by room temperature magnetic stirring. After the BDC dissolved completely in the mixed solvent, 2.88 mM (0.8374 g) of Nickel nitrate hexahydrate (Ni(NO_3_)_2_·6H_2_O) (SRL Chemical, 98%) was added to the previous mixture and dissolved by magnetic stirring. Then the final mixture was homogenized by ~30 min of ultrasonication followed by ~10–15 min magnetic stirring. It was then poured into a 100 mL Teflon-lined stainless steel autoclave, and the autoclave was kept inside a pre-heated oven at 125 °C for 15 h. Then the autoclave was taken out of the oven and allowed to cool down to room temperature. The precipitate was collected by centrifugation process. During centrifugation, it was washed with absolute ethanol three times. The precipitate was then dried at 50 °C for ~5 h inside an oven, followed by mild grinding to get the final Ni-MOF powder.

### 2.2. Fabrication of the Composite Films and Energy Harvesting Devices

To prepare the PVDF-based composite films, 8 wt% (with respect to solvent) of PVDF powder (Sigma Aldrich, Saint Louis, MO, USA, average molecular weight ~534,000 by GPC) was first dissolved in polar DMF solvent (inside a capped glass bottle) by magnetic stirring for ~1 h at 40 °C of hot plate temperature. Then 0.5 wt% (with respect to PVDF) of Ni-MOF powder was added to the PVDF solution, and the mixed solution (inside a capped glass bottle) was kept for continuous magnetic stirring for ~36 h at room temperature. In between this long duration of magnetic stirring, 30 min of ultrasonication was also applied to the solution after each 12 h of stirring in order to homogenize it. 2 mL of this homogeneous solution was then poured into a small petri-dish of ~47 mm diameter. The petri-dish was then kept inside a leveled air oven at 85 °C for ~3 h for annealing and solvent evaporation. After that, it was allowed to cool down to room temperature, and the desired composite film (named NPVDF) with ~30 μm thickness was peeled off from the petri-dish. A film of only PVDF without any filler loading was also prepared by following the same route. For electrical characterization, the composite films were cut into small sizes, and both sides were painted with high-quality silver electrodes followed by curing at 65 °C (~30 min electrode curing for each side). The piezoelectric nanogenerator (PENG) devices were fabricated by sandwiching the composite films in between two adhesive aluminum (Al) electrodes of 2 × 3 cm^2^ size. On the other hand, for the development of piezo-tribo hybrid nanogenerator (HNG) devices via contact-separation mode, one side of the composite films was affixed by an adhesive aluminum electrode, and the other side was kept open. The second electrode was placed such that (by using a PET support structure) there exists a certain air gap (~2 cm) between that electrode and the open surface of the composite films [44]. In this case, also, the electrode size was kept the same as that for the piezoelectric devices. In both cases, the area of the used composite films was slightly higher than that of the electrodes in order to completely cover them (electrodes). The piezo-tribo hybrid devices comprising the PVDF and NPVDF films were named PVDFH and NPVDFH, respectively.

### 2.3. Characterization Techniques

In order to evaluate the structure and morphology of the synthesized Ni-MOF, X-ray Diffraction (XRD) (Rigaku, Tokyo, Japan), Scanning Electron Microscopy (SEM) (Zeiss, Germany), and Transmission Electron Microscopy (TEM) (JEOL) was utilized. The same SEM instrument was also utilized in order to investigate the morphology of the composite films and the filler distribution within them. The elemental characteristics were investigated by Energy Dispersive X-ray (EDX) spectroscopy attached to the SEM instrument. The polarity of the composite films was investigated by using the Fourier Transform Infrared (FTIR) spectroscopy (PerkinElmer, Waltham, MA, USA). The mechanical strength of the composite films was evaluated by using a Universal Testing Machine (UTM) (SHIMADZU, Suzhou, Jiangsu, China) by applying a 6 mm/min strain rate in tensile mode. In order to explore the energy storage characteristics of the composite films, their dielectric properties were investigated by using an Impedance Analyzer (WAYNE KERR 6500B, West Sussex, UK). For this purpose, their electric field-dependent polarization loops (*P*-*E* loops) were also evaluated by using a custom-designed modified Sawyer-Tower circuit equipped with a function generator (Tektronix, AFG3022B, Beaverton, OR, USA), high voltage amplifier (Trek, PD05034, Lockport, NY, USA) and electrometer (KEITHLEY, 6517B, Beaverton, OR, USA) and interfaced with LabVIEW software (2017, Version: 17.0). The output voltages from the PENGs and HNGs (upon the application of repeated mechanical force on them) were recorded by using a digital storage oscilloscope (KEYSIGHT, DSOX1102G, Wokingham, UK). The output short-circuits current was measured by using a Precision source meter (Agilent B2902A, Santa Clara, CA, USA). The average value of the input repeated mechanical force was measured by using a pressure sensor (SHIMADZU, AUX220 weighing machine, Kyoto, Japan).

## 3. Results and Discussion

### 3.1. Structure and Morphology of the Synthesized Ni-MOF

**Figure 1:**
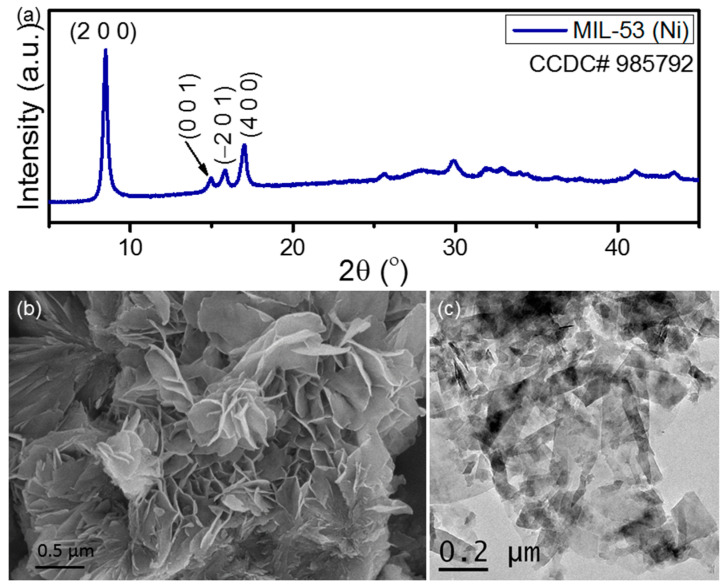
(**a**) XRD pattern, (**b**) SEM micrograph, and (**c**) bright field TEM image of the synthesized Ni-MOF nanosheets.

Before incorporating the synthesized fillers into the PVDF matrix, their structural and microstructural properties have been investigated here. Figure 1a presents the XRD pattern of the synthesized Ni-MOF powder. The XRD pattern has been found to be matched well with the XRD pattern of MIL-53 (Ni) powder (CCDC# 985792) reported earlier [45,46]. Thus, the MIL-53 structure of the synthesized Ni-MOF with *C2*/*m* space group has been confirmed from the XRD characterization. On the other hand, the morphology of the synthesized Ni-MOF powder has been investigated by using SEM and TEM characterization and presented in Figure 1b and Figure 1c, respectively. The highly 2D morphology of the Ni-MOF nanosheets can be clearly observed from these images. The average thickness of the 2D nanosheets has been found to be ~15 nm. On the other hand, the surface diameter/length of the nanosheets have shown a wide variation in their values, starting from ~150 nm to a couple of micrometers. However, the majority of the nanosheets exhibit an average diameter/length of ~300–350 nm. This result clearly proves the highly 2D feature of the synthesized Ni-MOF nanosheets. Thus, the highly 2D nature of this filler is expected to tune the polarity of the PVDF matrix very significantly by improving the number of active sites for interfacial interaction.

### 3.2. Microstructural and Elemental Characterizations of the Composite Films

**Figure 2:**
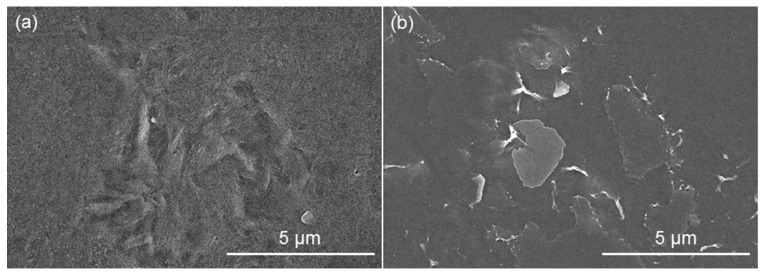
SEM image of (**a**) PVDF and (**b**) NPVDF films.

**Figure 3:**
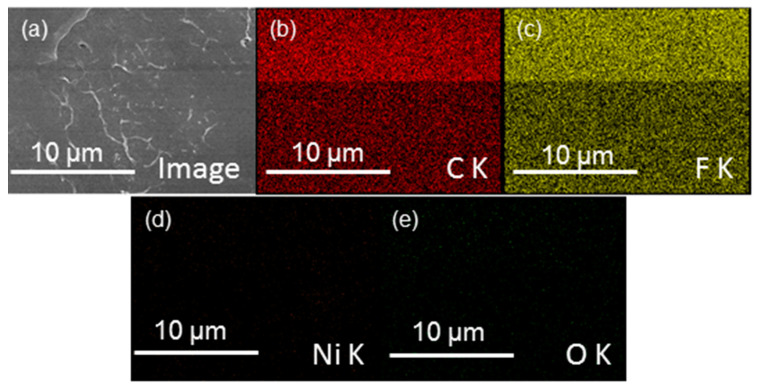
EDX mapping of (**a**) a random portion of the NPVDF film showing the existence of the desired elements such as (**b**) carbon, (**c**) fluorine, (**d**) nickel, and (**e**) oxygen.

The SEM images of neat PVDF and NPVDF films are presented in Figure 2a and Figure 2b, respectively. The distribution of 2D fillers within the PVDF matrix can be clearly observed from the SEM image of NPVDF. It is obvious that the appearance of fillers in the SEM image of NPVDF has been found in very few places which are attributed to the ultralow filler concentration in the host polymer matrix. The occurrence of the desired elements within the NPVDF film has been confirmed from the EDX characterization (taken at a random place of the composite), as presented in Figure 3. The amounts of different elements within both the PVDF and NPVDF films are presented in Table 1. Though hydrogen could not be detected through EDX characterization, the sum of the amount of nickel and oxygen within the NPVDF film has been found to be in an almost desired ratio. The increase in the relative amount of carbon for NPVDF film compared to that of PVDF also confirms the incorporation of Ni-MOF (MIL-53(Ni)) within PVDF. Thus, the expected filler distribution and elemental purity of the fabricated samples have been justified.

### 3.3. Analysis of Polarity

**Figure 4:**
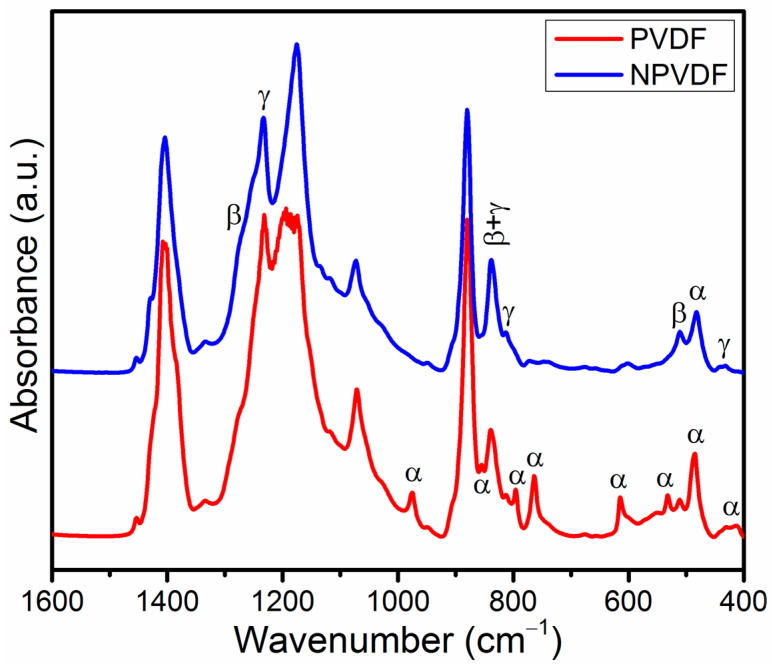
FTIR absorption spectra of PVDF and NPVDF films within the wide wavenumber region of 1600–400 cm^−1^.

As the Ni-MOF nanosheets exhibit high 2D morphology, they are expected to interact strongly with the PVDF matrix due to the availability of their higher amount of active sites. Fillers with high aspect ratios have also been previously reported to strongly tune the non-polar chain conformation of PVDF into its polar form [24]. This phase transformation commonly plays a very crucial role in determining the output functional performances of PVDF-based composites. Therefore, prior to the investigation of the electrical and electronic properties of the fabricated PVDF and NPVDF films, their detailed phase analysis is necessary. In order to study this phase analysis, detailed FTIR characterization and corresponding analysis were performed here. Figure 4 presents the FTIR absorption spectra of the fabricated PVDF and NPVDF films within a wide wavenumber region of 1600–400 cm^−1^. Neat PVDF exhibits strong absorption bands at 410, 484, 532, 615, 764, 796, 855, and 976 cm^−1^, which correspond to the non-polar α chain conformation of PVDF [47]. Considerable absorption bands at 838 cm^−1^ correspond to polar β and γ phase (combined) and at 1233 cm^−1^ correspond to γ phase, and very weak absorption bands at 510 and 1277 cm^−1^ correspond to β phase and at 811 cm^−1^ corresponding to γ phase have also been observed for this sample [47]. On the other hand, all the absorption bands corresponding to the non-polar α phase (except the 484 cm^−1^ band) have been found to be disappeared for the NPVDF film, and the intensity of all the absorption bands corresponding to polar β and γ phases for this film increases significantly compared to that of neat PVDF. This result clearly suggests that the slight loading of 2D Ni-MOF (0.5 wt% only) into the PVDF matrix strongly helps in transforming its energetically most favorable non-polar TG^+^TG^−^ chain configuration (α phase) into polar TTTT (β phase) and T_3_G^+^T_3_G^−^ (γ phase) arrangement which is attributed to the strong interfacial interaction of 2D Ni-MOF particle surfaces with PVDF dipoles [11,13,47].

Apart from the above understanding, it is a very common practice to quantitatively determine the number of polar phases in PVDF-based composite films by using the well-known Beer-Lambert law given by Equation (1) [48].

(1) $$F\left(EA\right)=\frac{A_{EA}}{\left(\frac{k_{840}}{k_{764}}\right)A_{NEA}+A_{EA}}\times 100\%$$

*F*(*EA*), *A_EA_,* and *A_NEA_* in the above equation represent the percentage of the total electroactive phase, the intensity of the electroactive 840 cm^−1^ band, and the intensity of the non-electroactive 764 cm^−1^ band, respectively. The absorption coefficients corresponding to the 840 and 764 cm^−1^ band with respective values of 7.7 × 10^4^ and 6.1 × 10^4^ cm^2^ mol^−1^ are represented by *k*_840_ and *k*_764_, respectively. The values of *F*(*EA*) calculated by using the above equation have been found to be ~55.1% and 84.6%, respectively, for PVDF and NPVDF samples. This result confirms the huge phase transformation of PVDF caused by ultralow Ni-MOF filler loading. The high value of *F*(*EA*) of NPVDF film proves the ultrahigh polarity of this film. The amounts of polar phases of some other similar kinds of samples are compared in Table 2 with the value obtained for NPVDF film in the present work. This value has been found to be within the comparable range with others. In most cases, comparably higher amounts of fillers have been incorporated into the PVDF matrix in order to achieve high polarity. Thus, the appearance of the high polarity of PVDF with only 0.5 wt% 2D Ni-MOF loading may be very advantageous for various functional applications, as described earlier.

**Figure 5:**
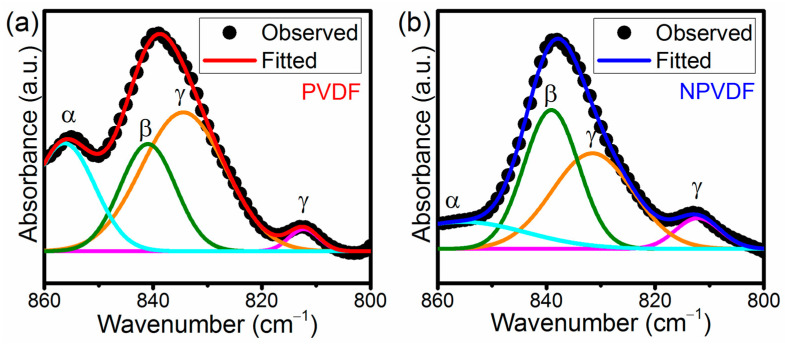
Gaussian curve fitting of the FTIR absorption spectra around 840 cm^−1^ band corresponding to (**a**) PVDF and (**b**) NPVDF samples. (The deconvoluted absorption peaks at 855, 840, 833 and 811 cm^−1^ corresponding to α, β, γ and γ phases, respectively, are presented in Cyan, Olive, Orange and Magenta colours.).

Not only the overall non-polar to polar phase transformation but the improvement of β phase relative to that of γ also plays a very important role in enhancing the piezoelectric performance of the resulting PVDF-based composites due to the unique all-trans chain conformation of the former (β phase) that exhibits the highest polarity compared to that of all other chain conformations. The idea about individual phase content can be evaluated from the FTIR absorption spectra through the Gaussian curve fitting of the absorption band around 840 cm^−1^ and by determining the area under the required deconvoluted peaks [24]. Figure 5a,b presents the Gaussian curve fitting of the 838 cm^−1^ absorption band of PVDF and NPVDF films, respectively. Upon this curve fitting, two distinct peaks at 840 and 833 cm^−1^ of wavenumber have clearly appeared for both the samples, which correspond to β and γ phases, respectively. These deconvoluted peaks are of particular interest in order to evaluate the amounts of individual phases. If *A_β_* and *A_γ_* be the integrated area under the deconvoluted 840 and 833 cm^−1^ peaks, respectively, then the individual β phase percentage (*F*(*β*)), individual γ phase percentage (*F*(*γ*)), the relative amount of β phase with respect to γ (*F*(*βγ*)) and relative amount of γ phase with respect to β (*F*(*γβ*)) can be calculated by using Equations (2)–(5), respectively, as given below.

(2) $$F\left(\beta \right)=F\left(EA\right)\times \frac{A_{\beta }}{A_{\beta }+A_{\gamma }}$$

(3) $$F\left(\gamma \right)=F\left(EA\right)\times \frac{A_{\gamma }}{A_{\beta }+A_{\gamma }}$$

(4) $$F\left(\beta \gamma \right)=\frac{F\left(\beta \right)}{F\left(\beta \right)+F\left(\gamma \right)}\times 100\%$$

(5) $$F\left(\gamma \beta \right)=\frac{F\left(\gamma \right)}{F\left(\beta \right)+F\left(\gamma \right)}\times 100\%$$

The values of these parameters for both the samples, as obtained from the Gaussian curve fitting, are presented in Table 3, which clearly suggests that there is a significant enhancement of β phase content of PVDF relative to the γ phase content after ultralow concentration of 2D Ni-MOF loading. Along with the improved overall polarity, this enhancement of individual β phase content in NPVDF film compared to that of PVDF suggests that their dipolar polarity and piezoelectric performance will also be enhanced.

### 3.4. Mechanical and Electrical Strengths

**Figure 6:**
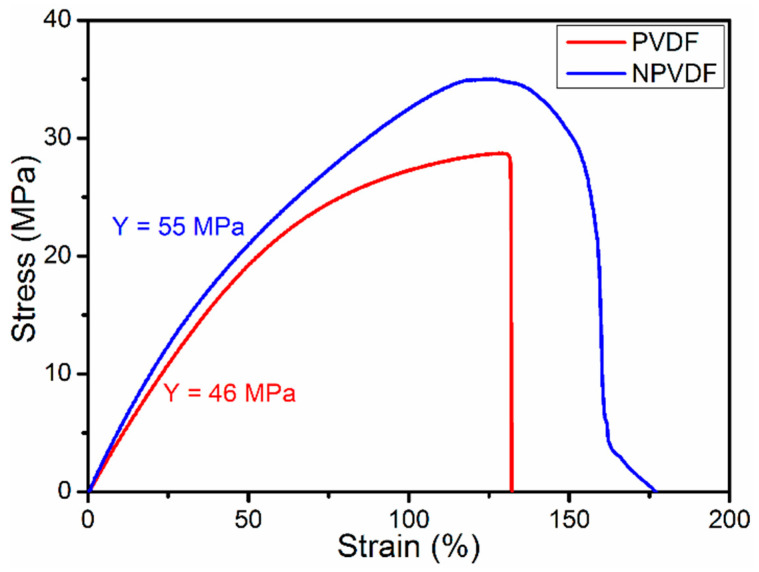
Stress-strain curves of PVDF and NPVDF films.

The reduction in mechanical and electrical strength of PVDF with increased concentration of filler loading is a very common phenomenon [43,52]. But both of these parameters strongly affect the output functional properties of dielectric materials. Therefore, one of the key ideas of selecting a low concentration of filler loading was to fabricate the functional film in such a way that its mechanical and electrical strength does not reduce significantly. Figure 6 presents the stress—strain curves (tensile mode) of PVDF and NPVDF films. Interestingly, the elongation/stretchability (maximum strain up to breakdown point) of NPVDF film has been found to be significantly higher (~159%) compared to that of the pristine PVDF film (~132%), which is directly attributed to the stress concentration effect induced by MOF loading within the PVDF matrix leading to an improved degree of crystallinity [39]. The calculated Young’s modulus of NPVDF film has also been found to be increased to ~55 MPa from a value of ~46 MPa for pristine PVDF. The Young’s modulus, along with the piezoelectric coefficient, renders a very strong effect on the electric charge separation (Di) activity of this type of film which can be expressed by Equation (6) as given below [39].

(6) $$D_{i}=d_{ij}\sigma_{j}=d_{ij}Y_{kj}\varepsilon_{k}$$

The *d_ij_*, *σ_j_*, *Y_kj_,* and *ε_k_* in the above equation represent the piezoelectric coefficient, applied stress, Young’s modulus, and applied strain, respectively. Thus, the increase in Young’s modulus of PVDF after MOF loading into it suggests that the energy harvesting performance will also be improved.

Capacitive energy storage performance, on the other hand, strongly depends on the electrical breakdown strength (BDS) along with the dielectric permittivity. If *k_eff_* is the effective dielectric permittivity, *ε*_0_ is the free space permittivity, and *E_b_* is the BDS, then the energy storage density (*U_d_*) of any linear dielectric capacitor can be expressed by Equation (7) as depicted below [42].

(7) $$U_{d}=\frac{1}{2}k_{eff}\varepsilon_{0}E_{b}^{2}$$

Though PVDF is a non-linear dielectric material and its *U_stored_* can not be calculated by using the above equation, it can be easily inferred that the BDS has the most important effect on its energy storage performance. The parameters related to the energy storage performance of PVDF-based composites are commonly calculated from their polarization (*P*)—electric field (*E*) hysteresis loops (*P-E* loops). In the present case, the BDS of PVDF and NPVDF films has been found to be ~715 and 920 kV/cm. This is a very interesting result in the contest of energy storage performance. Commonly, when a higher amount of filler is incorporated into PVDF film, the BDS of the resulting composite system decreases [53,54,55]. At an optimized filler concentration, especially for 2D fillers like Boron Nitride Nanosheets (BNNS) and MoS_2_, the BDS has been found to be improved in some previous reports [54,55]. Here in the present work, also, small loading (0.5 wt%) of 2D Ni-MOF was able to significantly improve the BDS up to a certain amount. This improvement is probably attributed to the homogeneous filler distribution, less filler agglomeration owing to low content filler loading, and the trapping of charge carriers in the interface region restraining charge migration [56,57]. The high concentration filler loading causes the reduction in the relative distance between adjacent filler particles causing easy charge migration, which is hindered for a lower amount of filler loading [57]. Thus, the high BDS of NPVDF compared to that of the PVDF is justified. Moreover, the strong interfacial interaction of 2D Ni-MOF particle surface with PVDF dipoles helps in reducing the small voids within the composite film, which is also advantageous for improving the BDS [56]. In this regard, it is to be mentioned that the obtained values of BDS for both the PVDF and NPVDF films have been found to be much lower than that of the reported values for similar samples, which is attributed to the following factors. Actually, in order to achieve high BDS and hence the energy storage performance, it is a very common trend to follow some post-casting (solvent casting) techniques like thermal quenching, hot-pressing, etc., during sample fabrication which can significantly reduce the microscopic voids and defects. Here in the present work, no such techniques were utilized, which has resulted in low BDS. Thus the obtained result only infers the effect of the material only (positive effect of low-content 2D Ni-MOF in improving the BDS of PVDF). These properties can be obviously improved further by following the above-mentioned strategies.

### 3.5. Dielectric, Ferroelectric, and Energy Storage Properties

**Figure 7:**
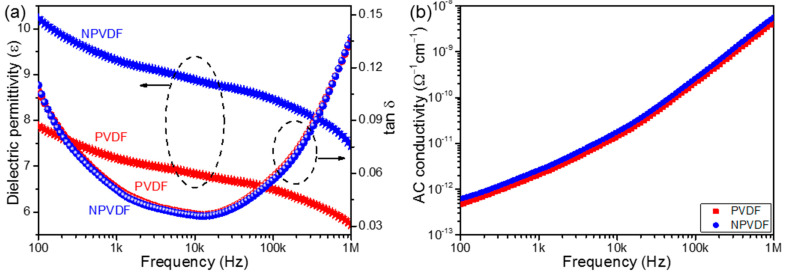
Frequency-dependent (**a**) dielectric permittivity and tan δ and (**b**) AC conductivity of the fabricated composite films.

As stated earlier, the energy storage performance of PVDF-based composite films depends on their dielectric properties and BDS and can be calculated from their ferroelectric *P-E* loops. Therefore, it is essential to study the dielectric and ferroelectric properties of the fabricated PVDF and NPVDF films. Figure 7a presents the frequency-dependent dielectric permittivity and loss tangent (*tan δ*) of these films, and their frequency-dependent AC conductivities are illustrated in Figure 7b. The significant enhancement of dielectric permittivity of NPVDF compared to that of PVDF in the entire frequency region confirms the enhancement in dipolar polarity caused by the interfacial interaction between the 2D Ni-MOF filler particles and PVDF dipoles [58]. The slight increase in the steepness of the dielectric permittivity curve of NPVDF in the low-frequency region and the slight increase in the low-frequency tan δ compared to those of PVDF confirms the slight increase in space charge polarization, too, which is attributed to the slightly enhanced conducting pathway due to filler addition [58]. The marginal increase in AC conductivity of PVDF after 0.5 wt% 2D Ni-MOF loading (Figure 7b) also supports this fact. The values of *tan δ* for both samples have been found to be low (<0.14) in the entire frequency region. The marginal reduction in the value of *tan δ* for the NPVDF sample compared to that of PVDF is attributed to the similar factors as described while explaining the BDS results. The huge improvement in the dielectric permittivity of NPVDF suggests that the energy storage density may also be improved compared to that of PVDF.

**Figure 8:**
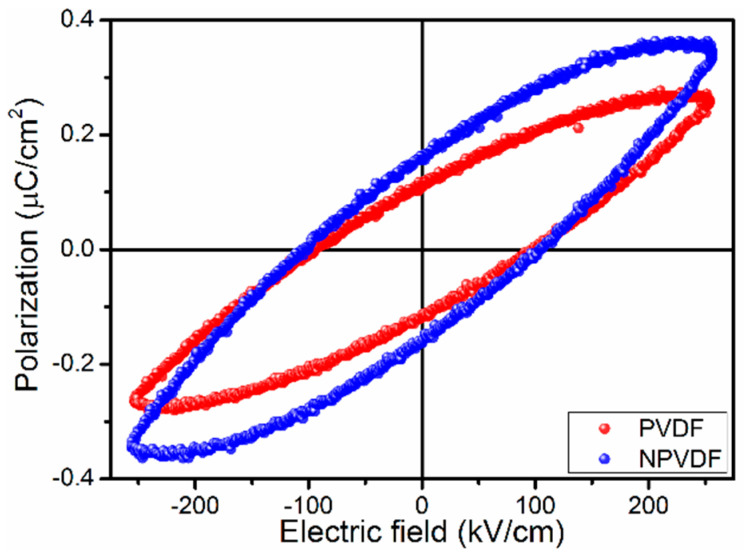
*P-E* hysteresis loops of PVDF and NPVDF films.

Figure 8 presents the *P-E* hysteresis loops (measured at 1 Hz of the triangular wave) of the fabricated composite film. The highly lossy nature of the obtained *P-E* loops suggests the presence of space charge polarization along with dipolar polarization (as confirmed earlier) [59]. In this regard, it is to be mentioned that this type of lossy *P-E* loop is not suitable for energy storage purposes. They commonly render very low values of efficiency. For good values of energy storage parameters (density and efficiency), slim *P-E* loops are desired. Thus the obtained *P-E* loops can only give ideas about the effect of the material only. In order to achieve good energy storage performance, the post-casting treatments as described earlier and the electrode optimization can be followed. However, the obtained *P-E* loops clearly suggest the increase in polarization of PVDF after a small amount of 2D Ni-MOF incorporation into it. This increased polarization value suggests that the used filler is suitable to enhance the capacitive energy storage performance of PVDF. On the other hand, the high value of BDS of the NPVDF film compared to that of PVDF is also very advantageous for higher energy storage performance. However, in order to show the effect of the materials, the *P-E* loops of both of the fabricated samples have been compared at a moderate applied electric field of 250 kV/cm. The calculated energy storage density (by calculating areas under desired portion *P-E* loops) [42] of NPVDF increased to ~18 mJ/cm^3^ with 25% efficiency from a value of ~12 mJ/cm^3^ with 25% efficiency. It is true that the obtained values of energy storage parameters are not high enough to be comparable with PVDF-based superior energy storage devices. This could be resolved further by following the strategies mentioned above. However, the idea about the effect of 2D Ni-MOF addition in the PVDF matrix could be obtained from the observed *P-E* loops and calculated storage parameters. The low amount of 2D Ni-MOF addition has been found to be very helpful in improving the energy storage density of PVDF without reducing the efficiency, and hence, this result can be considered to be very good regarding flexible energy storage device applications.

### 3.6. Mechanical Energy Harvesting Performances

**Figure 9:**
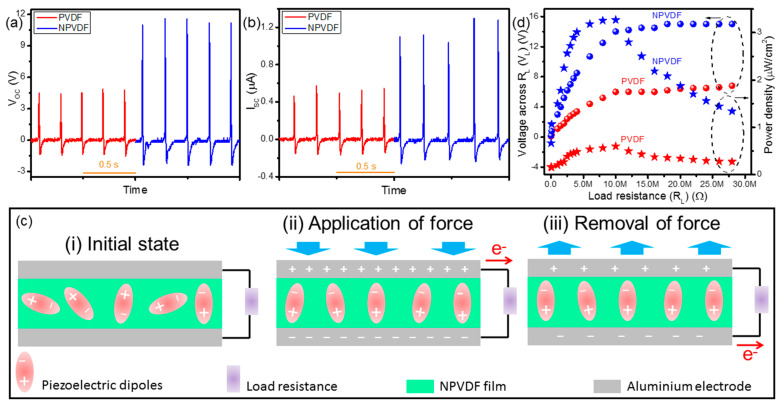
Output (**a**) *V_OC_* and (**b**) *I_SC_* from the PVDF and NPVDF-based PENGs. (**c**) Mechanism of piezoelectric energy harvesting. (**d**) *R_L_*-dependent *V_L_* and power density of the fabricated PENGs.

The enhancement of polarity and Young’s modulus of PVDF caused by Ni-MOF loading, as described earlier, suggested the improvement of its piezoelectric behavior [39]. Therefore, the piezoelectric energy harvesting performance of PVDF is expected to be significantly enhanced after the said filler loading. In order to investigate this phenomenon, the PENG devices comprising of PVDF and NPVDF films have been acted upon by an optimized periodic mechanical force (human finger tapping) of ~4 kPa at 5 Hz frequency, and the corresponding output electrical signals in terms of peak-to-peak open circuit voltage (*V_OC_*) have been recorded. As shown in Figure 9a, the output *V_OC_* from PVDF and NPVDF-based PENGs has been found to be ~5.5 and 15 V, respectively. Figure 9b presents the output short circuit current (*I_SC_*) signal from these devices upon similar applied mechanical stimuli. The values of peak-to-peak *I_SC_* from PVDF and NPVDF-based PENGs have been found to be ~0.6 and 1.5 μA, respectively. This huge enhancement of output electrical signal of NPVDF-based PENG compared to that of PVDF-based PENG is nothing but due to the significantly enhanced polarity and Young’s modulus of the former compared to that of the latter [39]. Both devices work on the basis of a similar mechanism, as shown in Figure 9c. The occurrence of output electrical signals from these types of un-poled PENGs is mainly driven by the stress-induced poling effect [50,60]. At the initial state (step-i), when there is no applied force, the polar dipoles within the films are oriented randomly, which results in zero piezoelectric potential. In the next step (step-ii)), when the applied vertical compressive force comes into play, the piezoelectric potential is abruptly generated within the dipoles, and the dipoles orient themselves in a single direction along the applied force due to their high directionality caused by stress-induced poling effect [50,60]. As a result of this fact, the piezoelectric potential is developed across the thickness of the composite films. To screen this piezoelectric potential, opposite charges are accumulated in the electrodes resulting in the flow of output current through the external load resistance [60]. Upon the removal of the applied force (step-iii)), the said piezoelectric potential diminishes, which results in the flow of the accumulated charges in the backward direction relative to that of the previous step. Therefore, with the continuation of repeated applied force and its removal, the output AC signal, as depicted in Figure 9a,b is generated. Though both the PVDF and NPVDF-based PENGs work on the basis of this mechanism, the higher output performance of the NPVDF-based PENG is directly attributed to the availability of a higher amount of polar dipoles within this film compared to that of pristine PVDF (explained earlier) [61]. Thus, the NPVDF film is expected to be more useful for real-life device applications. In order to study the applicability of these types of nanogenerator devices, it is a very common approach to study the output electricity with respect to the varied load resistance. Figure 9d presents the output voltage (*V_L_*) and power density (*P*) of both the fabricated PENGs across a wide variation of load resistance (*R_L_*). The *V_L_* first increases abruptly with the increase in *R_L,_* and then it almost saturates (becomes equal to the *V_OC_* of the respective device) after a certain high value of *R_L_* (~10 MΩ). On the other hand, *P* first increases with the increase in *R_L_* up to a certain maximum value and then decreases with the increase in *R_L_,* which is attributed to the well-known maximum power transfer theorem [62]. The maximum value of *P* has been found to be ~0.6 and 3.26 µW/cm^2^ (with load current (*I_L_*) ~0.6 and 1.4 µA) for PVDF and NPVDF-based PENGs (at 10 MΩ), respectively, which proves the superiority of the NPVDF-based device over PVDF-based PENG to drive low-power-consuming small electronic devices.

Apart from piezoelectric energy harvesting, PVDF-based composite films can be applied for triboelectric energy harvesting too. Triboelectric energy harvesting mainly relies on the mechanism of contact electrification and electrostatic induction [63]. Therefore, the surface roughness, the difference between the positions of two used triboelectric layers (for contact-separation modes) in the triboelectric series, and many other factors determine the performance of triboelectric nanogenerators. All these mentioned factors directly or indirectly affect the surface triboelectric charge density (*σ*), which directly determines the output *V_OC_* of triboelectric nanogenerators by following Equation (8) as depicted below [63,64].

(8) $$V_{OC}=\frac{\sigma x\left(t\right)}{\varepsilon_{0}}$$

Here, *ε*_0_ and *x*(*t*) represent the free space permittivity and distance between two triboelectric layers (one dielectric layer and another metal electrode in the present case of device design), respectively. If the distance between two layers, the relative permittivity of the dielectric layer, the work function of the upper metal electrode, and the work function of lower dielectric material be represented by *d*, *ε_r_*, *Φ_M_,* and *Φ_D_*, respectively, the surface triboelectric charge density (*σ*) can be expressed by the Equation (9) as given below [63,64].

(9) $$\sigma =2\varepsilon_{0}\varepsilon_{r}\left(\frac{\Phi_{M}-\Phi_{D}}{d}\right)$$

**Figure 10:**
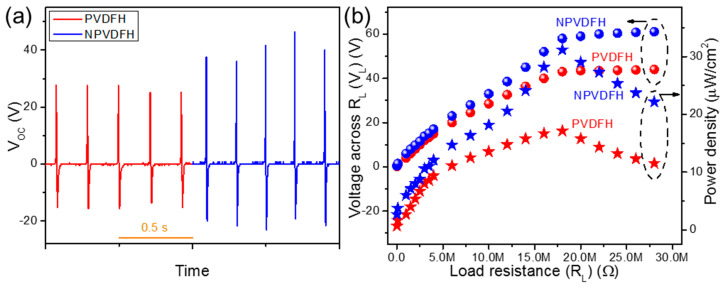
(**a**) Output *V_OC_* and (**b**) *R_L_*-dependent *V_L_* and power density of the fabricated PVDFH and NPVDFH devices.

Based on all of the above facts, the performance of a triboelectric nanogenerator can be considered to be greatly dependent on the dielectric permittivity of the used triboelectric layer. In the present case, the dielectric permittivity of NPVDF has been found to be higher than that of PVDF. Moreover, PVDF resides almost near the bottom of the triboelectric series and is an extremely negative triboelectric material [65]. On the other hand, being an extremely positive triboelectric material, aluminum (Al) resides almost near the top of the triboelectric series [65]. Therefore, the triboelectric device comprising of NPVDF film and Al electrode via contact-separation mode is expected to show enhanced performance compared to that of pristine PVDF. Though the good dielectric and surface properties of triboelectric layers are sufficient for excellent output electricity generation [66], the presence of piezoelectricity in the used dielectric layer may add an extra effect on the output performance of the final device. In the present case, the NPVDF film has been found to show higher piezoelectric energy harvesting activity compared to that of pristine PVDF. Thus, considering all of these advantages, the NPVDF-based piezo-tribo hybrid nanogenerator device (NPVDFH) is expected to show improved performance compared to that of PVDF-based HNG (PVDFH). Figure 10a shows the output *V_OC_* signals from PVDFH and NPVDFH devices upon the same applied input mechanical stimuli (~4 kPa, 5 Hz) as that for piezoelectric devices. The average values of *V_OC_* from these two devices have been found to be ~43 and 61 V, respectively. The *R_L_*-dependent *V_L_* and *P* curves (Figure 10b) follow a similar trend as that for piezoelectric devices with a maximum value of *P* at 18 MΩ of load resistance. The maximum values of *P* from PVDFH and NPVDFH have been found to be ~17 and 31 µW/cm^2^ with *I_L_* ~2.4 and 3.2 µA, respectively, at 18 MΩ of *R_L_*. The higher output performance of NPVDFH device compared to that of PVDFH is directly associated with its higher dielectric permittivity and piezoelectric characteristics, as observed earlier. The value of *P* from the NPVDFH device has been found to be within the comparable range (even a bit higher for some cases) with that of some other similar kinds of devices (Table 4) reported earlier. Thus, this result clearly proves the superiority of NPVDFH devices for real-life energy harvesting applications. The improved performance of the fabricated HNGs compared to that of the corresponding PENGs is directly associated with the combined effect of piezoelectricity and contact electrification [59,63], as described below.

**Figure 11:**
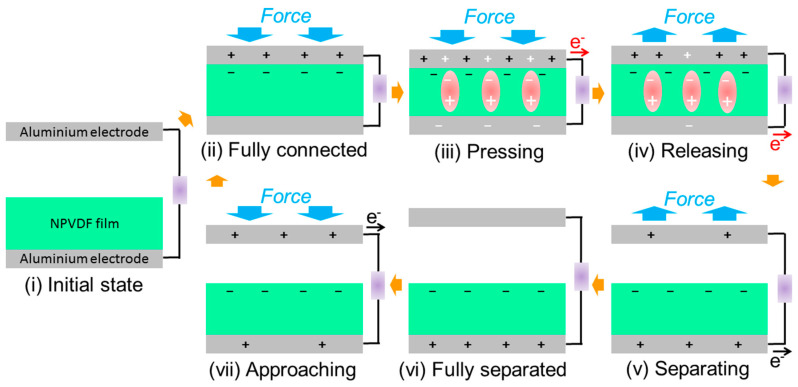
Schematic illustration of the proposed mechanism of piezoelectric-triboelectric hybrid energy harvesting. (Charges related to the piezoelectric effect are represented in white color, and those related to the triboelectric effect are represented in black color. The direction of electron flow due to the piezoelectric effect is shown in red color arrow, and that due to the triboelectric effect is shown in black color arrow. All other colors have the same notation as that for the piezoelectric mechanism shown in Figure 9. For the sake of simplicity, the piezoelectric dipoles are not shown in all the steps except step-iii and iv.).

Unlike the mechanism of the conventional dielectric film—metal electrode-based triboelectric nanogenerators, the piezoelectric effect also comes into play here in the energy harvesting mechanism (Figure 11) for the desired performance enhancement [72]. In the initial state (step-i), when there is no applied force, no triboelectric or piezoelectric charges are present there, resulting in no output electricity. In the next step (step-ii), when the lower PVDF-based film and the upper Al electrode just come to contact with each other upon the application of mechanical force, negative and positive charges are generated on the respective surfaces due to their negative and positive tribo-polarity, respectively. After this contact, the applied force helps in compressing the PVDF-based layer vertically (step iii), which results in the appearance of piezoelectric potential and corresponding current flow through the external load resistance (similar to that of piezoelectric energy harvesting mechanism). Now, when the applied force is removed, this compression is first released (step-iv), resulting in the piezoelectric current flow in the opposite direction. After the compression is released, the upper Al electrode starts separating from the lower PVDF-based layer (step-v), and hence, the triboelectric effect comes into the picture through the generation of positive triboelectric charges in the bottom electrode due to the effect of electrostatic induction of negative charges on the film surface. With the complete removal of the applied force, when the upper Al electrode is completely separated from the lower PVDF-based layer (step-vi), electrical equilibrium is reached, which results in no output electrical signal. When the external mechanical force is started to be applied again, the two layers start approaching each other (step-vii), which results in the generation of positive tribo-charges on the upper electrode by the effect of electrostatic induction. As a result of this fact, triboelectric current flows in the opposite direction relative to that of the previous direction. After this, the two layers again come into contact (step ii), and a similar effect repeats. This process continues with the repeated application and removal of input mechanical stimuli, which results in the generation of the output AC signal. Steps vii, ii, and iii together create a one-half cycle of the output signal. On the other hand, the combined effect of steps iv and v is responsible for another half cycle. This beautiful mechanism helps in improving the output performance of the HNGs compared to that of the PENGs, which makes them suitable for several real-life applications.

**Figure 12:**
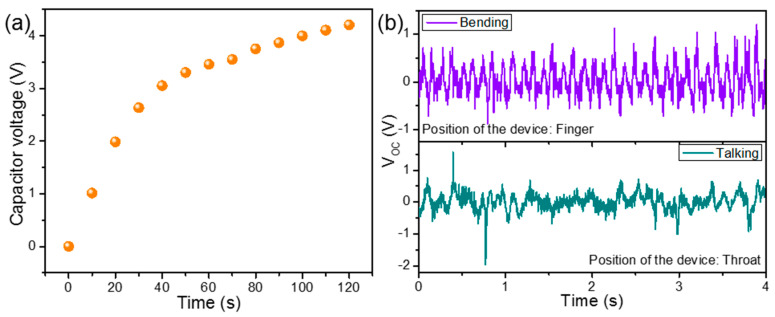
(**a**) Capacitor charging by using the rectified electricity from NPVDFH device upon repeated human finger tapping on it for a certain period of time. (**b**) Output *V_OC_* from the NPVDFH device during various human body movement activities (after attaching the device with respective body parts).

Both of these types of PENGs and HNGs have been previously explored in a wide variety of real-life applications, such as human motion interactive ambient mechanical energy harvesting, powering small electronic devices, and self-powered vibration sensing [63,73]. Due to the obtained high output performance, some real-life applications of the NPVDFH device have also been demonstrated here for the proof of concept. The device was first connected across a commercial 10 µF capacitor via a bridge rectifier circuit and was acted upon by repeated finger tapping for 120 s. During this short period of finger tapping, the device was able to charge the capacitor up to ~4.2 V of DC (Figure 12a). This stored power in the capacitor can be used to drive various low-power-consuming electronic devices, which proves the ability of the device for power-source applications [63]. In order to demonstrate its self-powered sensing application, it has been first affixed between two fingers. The bending caused by the repeated relative movement of the fingers was sufficient to generate a certain output voltage (Figure 12b). In another experiment, the device was affixed to the human throat, and the output was recorded during random talking. A certain significant value of output signal has been achieved in this case, too (Figure 12b). These experiments demonstrate the usability of the fabricated device in energy harvesting from human motion interactive ambient vibrations, self-powered vibration sensing, and healthcare monitoring [73]. These types of fabricated devices can be utilized in a variety of other real-life applications too, which are out of the focus of the present work and hence have not been shown here.

## 4. Conclusions

Highly 2D Ni-MOF nanosheets with MIL-53 structure were synthesized here via a simple hydrothermal route. Low concentrations (0.5 wt%) of these nanosheets were then incorporated into the PVDF matrix, and the corresponding composite films were fabricated through the conventional solvent casting technique. Both the electrical and mechanical strength of low-content filler-loaded PVDF film (NPVDF) were found to be improved significantly (BDS~920 kV/cm and strain/stretchability~159%) comped to that of pristine PVDF (BDS~715 kV/cm and strain/stretchability~132%). This result justified the motivation of selecting low-concentration filler loading. Furthermore, the polarity, dielectric permittivity, and ferroelectric polarization of PVDF were also significantly improved even after this low-concentration filler loading. The value of *F*(*EA*) and dielectric permittivity of PVDF were found to be increased from ~55% and 7.1 (at 1 kHz) to ~85% and 9.3 after the mentioned amount of 2D Ni-MOF loading. These results were mainly attributed to the strong interfacial interaction between fillers and PVDF dipoles caused by the high surface area of the highly 2D shape of the filler. As a result of these facts, the NPVDF film showed better dielectric energy storage and piezoelectric energy harvesting performances compared to those of PVDF. Moreover, the mechanical energy harvesting performance of both composite films was significantly improved further by the fabrication of their piezo-tribo hybrid structure in contact-separation mode. This was attributed to the combined effect of piezoelectricity and triboelectrification, which has been fruitfully explained here. The hybrid device comprising of NPVDF film was able to render very high output power density (~31 μW/cm^2^), which was very useful for capacitor charging (power source) and human motion interactive self-powered vibration sensing.

## Figures and Tables

**Table 1:** Amounts of desired elements within the PVDF and NPVDF films as observed from the EDX characterization.

Sample	PVDF				NPVDF			
Elements	Carbon	Fluorine	Nickel	Oxygen	Carbon	Fluorine	Nickel	Oxygen
Weight%	48.88	51.12	-	-	53.68	45.69	0.08	0.55
Atomic%	60.19	39.81	-	-	64.68	34.80	0.02	0.50

**Table 2:** Comparison of the amount of polar phase of NPVDF film with some other similar kinds of composites as reported earlier.

Sl. No.	Polymer Matrix	Filler Material	Filler Concentration	*F*(*EA*) (%)	Ref. No.
1.	PVDF	Bi_0.9_Ba_0.1_FeO_3_	7 wt%	82	[11]
2.	PVDF	ZnO nanorod	20 wt%	88	[24]
3.	PVDF	Mo_0.5_W_0.5_S_2_ nanosheet	~28 wt%	90	[28]
4.	PVDF	WS_2_ nanosheet	~0.25 wt%	85	[30]
5.	PVDF	Graphite platelet	0.5 wt%	95	[33]
6.	PVDF	[CdI_2_-INH=CMe_2_ (3D MOF)	1 wt%	98	[38]
7.	PVDF	CsPbBr_3_ nanorod	5 wt%	90	[49]
8.	PVDF	Ba_0.6_Sr_0.4_TiO_3_	20 wt%	90	[50]
9.	PVDF	PEG-modified ZnFe_2_O_4_	10 wt%	92	[51]
10.	PVDF	2D Ni-MOF	0.5 wt%	85	This work

**Table 3:** Individual polar phase contents in the fabricated composite films.

Sample Name	*F*(*EA*) (%)	*F*(*β*) (%)	*F*(*γ*) (%)	*F*(*βγ*) (%)	*F*(*γβ*) (%)
PVDF	55.1	26.29	28.80	47.72	52.28
NPVDF	84.6	42.20	42.40	49.88	50.12

**Table 4:** Comparison of output power density (*P*), load voltage (*V_L_*), and load current (*I_L_*) of the fabricated NPVDFH device (HNG) with some other similar kinds of devices reported earlier.

Sl. No.	Device Structure (Lower Layer/Upper Layer)	*P* (μW/cm^2^)	*R_L_* at *P* (MΩ)	*V_L_* (V)	*I_L_* (μA)	Ref. No.
1.	PVDF-TrFE+PEDOT:PSS/PI	1.28	10	15.6	~1	[67]
2.	PVDF+ZnO/PTFE	24.5	140	~78	-	[68]
3.	PVDF+BaTiO_3_/Nylon	22.5	100	160	~1	[69]
4.	PVDF+NaNbO_3_ (Polarised)/PTFE	22.6	100	160	-	[70]
5.	PVDF+Dopamine@SnO_2_/Paper	81	~100	~100	-	[71]
6.	PVDF+BCZT/Al	76.7	20	89.8	4.8	[59]
7.	PVDF+BiFeO_3_/Al	61	20	80	~4	[63]
8.	PVDF+Ni-MOF/Al	31	18	61	3.26	This work

## Data Availability

The data in this study will be made available upon request from the corresponding authors.

## References

[1] Maiti T.K., Singh J., Majhi J., Ahuja A., Maiti S., Dixit P., Bhushan S., Bandyopadhyay A., Chattopadhyay S. (2022). Advances in Polybenzimidazole Based Membranes for Fuel Cell Applications That Overcome Nafion Membranes Constraints. Polymer.

[2] Sardana S., Gupta A., Singh K., Maan A.S., Ohlan A. (2022). Conducting Polymer Hydrogel Based Electrode Materials for Supercapacitor Applications. J. Energy Storage.

[3] Darwish M.A., Zubar T.I., Kanafyev O.D., Zhou D., Trukhanova E.L., Trukhanov S.V., Trukhanov A.V., Henaish A.M. (2022). Combined Effect of Microstructure, Surface Energy, and Adhesion Force on the Friction of PVA/Ferrite Spinel Nanocomposites. Nanomaterials.

[4] Korolkov I.V., Zhumanazar N., Gorin Y.G., Yeszhanov A.B., Zdorovets M.V. (2020). Enhancement of Electrochemical Detection of Pb2+ by Sensor Based on Track-Etched Membranes Modified with Interpolyelectrolyte Complexes. J. Mater. Sci. Mater. Electron..

[5] Chen Y., Yang Y., Orr A.A., Makam P., Redko B., Haimov E., Wang Y., Shimon L.J.W., Rencus-Lazar S., Ju M. (2021). Self-Assembled Peptide Nano-Superstructure towards Enzyme Mimicking Hydrolysis. Angew. Chemie.

[6] Pei J.Y., Yin L.J., Zhong S.L., Dang Z.M. (2023). Suppressing the Loss of Polymer-Based Dielectrics for High Power Energy Storage. Adv. Mater..

[7] Yuan H., Chen Y., Lin R., Tan D., Zhang J., Wang Y., Gazit E., Ji W., Yang R. (2022). Modified Stranski-Krastanov Growth of Amino Acid Arrays toward Piezoelectric Energy Harvesting. ACS Appl. Mater. Interfaces.

[8] Jaffar S.S., Saallah S., Misson M., Siddiquee S., Roslan J., Saalah S., Lenggoro W. (2022). Recent Development and Environmental Applications of Nanocellulose-Based Membranes. Membranes.

[9] Mohammadpourfazeli S., Arash S., Ansari A., Yang S., Mallick K., Bagherzadeh R. (2023). Future Prospects and Recent Developments of Polyvinylidene Fluoride (PVDF) Piezoelectric Polymer; Fabrication Methods, Structure, and Electro-Mechanical Properties. RSC Adv..

[10] Panicker S.S., Rajeev S.P., Thomas V. (2023). Impact of PVDF and Its Copolymer-Based Nanocomposites for Flexible and Wearable Energy Harvesters. Nano-Struct. Nano-Objects.

[11] Sasmal A., Sen S., Devi P.S. (2019). Role of Suppressed Oxygen Vacancies in the BiFeO_3_ Nanofiller to Improve the Polar Phase and Multifunctional Performance of Poly(Vinylidene Fluoride). Phys. Chem. Chem. Phys..

[12] Chinya I., Sen S. (2017). Improved Dielectric and Touch Sensing Performance of Surface Modified Zinc Ferrite (ZF)/Polyvinylidene Fluoride (PVDF) Composite. Sens. Actuators A Phys..

[13] Sasmal A., Patra A., Devi P.S., Sen S. (2021). Hydroxylated BiFeO_3_ as Efficient Fillers in Poly(Vinylidene Fluoride) for Flexible Dielectric, Ferroelectric, Energy Storage and Mechanical Energy Harvesting Application. Dalt. Trans..

[14] Padurariu L., Horchidan N., Ciomaga C.E., Curecheriu L.P., Lukacs V.A., Stirbu R.S., Stoian G., Botea M., Florea M., Maraloiu V.A. (2022). Influence of Ferroelectric Filler Size and Clustering on the Electrical Properties of (Ag-BaTiO_3_)-PVDF Sub-Percolative Hybrid Composites. ACS Appl. Mater. Interfaces.

[15] Agayev F.G., Trukhanov S.V., Trukhanov A.V., Jabarov S.H., Ayyubova G.S., Mirzayev M.N., Trukhanova E.L., Vinnik D.A., Kozlovskiy A.L., Zdorovets M.V. (2022). Study of Structural Features and Thermal Properties of Barium Hexaferrite upon Indium Doping. J. Therm. Anal. Calorim..

[16] Jiang J., Li J., Zhang Y., Yuan Y., Liu X., Zuo P., Qian J., Zhuang Q. (2022). Tuning the Interfacial Insulating Shell Characteristics in CaCu_3_Ti_4_O_12_ Nanowires/Polyetherimide Nanocomposites for High-Temperature Capacitive Energy Storage. J. Mater. Chem. C.

[17] Trukhanov A.V., Almessiere M.A., Baykal A., Slimani Y., Trukhanova E.L., Timofeev A.V., Kostishin V.G., Trukhanov S.V., Sertkol M., Ul-Hamid A. (2023). Correlation between the Composition, Structural Parameters and Magnetic Properties of Spinel-Based Functional Nanocomposites. Nano-Struct. Nano-Objects.

[18] Kozlovskiy A.L., Shlimas D.I., Zdorovets M.V. (2021). Synthesis, Structural Properties and Shielding Efficiency of Glasses Based on TeO_2_-(1-x)ZnO-XSm_2_O_3_. J. Mater. Sci. Mater. Electron..

[19] Bhattacharya G., Das S., Venimadhav A. (2023). Study on the Synergistic Effects of Aqueous Rare Earth Salt and ZnO Nanoparticles on Poly (Vinylidene Fluoride) PVDF Based Self-Polarized, Electroactive Films. Curr. Appl. Phys..

[20] Zdorovets M.V., Kozlovskiy A.L., Shlimas D.I., Borgekov D.B. (2021). Phase Transformations in FeCo—Fe_2_CoO_4_/Co_3_O_4_-Spinel Nanostructures as a Result of Thermal Annealing and Their Practical Application. J. Mater. Sci. Mater. Electron..

[21] Mandal S., Hou Y., Wang M., Anthopoulos T.D., Choy K.L. (2023). Surface Modification of Hetero-Phase Nanoparticles for Low-Cost Solution-Processable High-k Dielectric Polymer Nanocomposites. ACS Appl. Mater. Interfaces.

[22] Zhou J., Zhou W., Yuan M., Dong X., Zhang J., Zhang X., Zhang Y., Chen X., Chen Y., Liu X. (2023). Significantly Suppressed Dielectric Loss and Enhanced Breakdown Strength in Core@Shell Structured Ni@TiO2/PVDF Composites. Nanomaterials.

[23] Mukherjee A., Dasgupta Ghosh B. (2023). Synthesis of Functionalized ZnO Nanoflake Loaded Polyvinylidene Fluoride Composites with Enhanced Energy Storage Properties. Polym. Compos..

[24] Pratihar S., Medda S.K., Sen S., Devi P.S. (2020). Tailored Piezoelectric Performance of Self-Polarized PVDF-ZnO Composites by Optimization of Aspect Ratio of ZnO Nanorods. Polym. Compos..

[25] Zhu M., Li G., Xu H., Xie H., Liao Y. (2023). Controllable Preparation of CaCu_3_Ti_4_O_12_ Nanowires and Its Strengthening Effect on High Dielectric Polymer Composites. J. Mater. Sci. Mater. Electron..

[26] Li G., Deng W., Li W., Yang M., Cui W. (2023). Synergistic Enhancement in Mechanical, Thermal, and Dielectric Properties of PANI@BT/PVDF Composites by Adding 2D Nanoplatelets. J. Appl. Polym. Sci..

[27] Jangra M., Thakur A., Dam S., Chatterjee S., Hussain S. (2023). Enhanced Dielectric Properties of MoS_2_/PVDF Free-Standing, Flexible Films for Energy Harvesting Applications. Mater. Today Commun..

[28] Bhattacharya D., Mukherjee S., Pal A.N., Mitra R.K., Ray S.K. (2022). Two-Dimensional MoxW1−xS2 Alloys for Nanogenerators Producing Record Piezo-Output and Coupled Photodetectors for Self-Powered UV Sensor. Adv. Opt. Mater..

[29] Wang B., Yin X., Peng D., Zhang Y., Wu W., Gu X., Na B., Lv R., Liu H. (2020). Highly Thermally Conductive PVDF-Based Ternary Dielectric Composites via Engineering Hybrid Filler Networks. Compos. Part B Eng..

[30] Bhattacharya D., Bayan S., Mitra R.K., Ray S.K. (2021). 2D WS2embedded PVDF Nanocomposites for Photosensitive Piezoelectric Nanogenerators with a Colossal Energy Conversion Efficiency of ∼25.6%. Nanoscale.

[31] Rajavel K., Luo S., Wan Y., Yu X., Hu Y., Zhu P., Sun R., Wong C. (2020). 2D Ti_3_C_2_Tx MXene/Polyvinylidene Fluoride (PVDF) Nanocomposites for Attenuation of Electromagnetic Radiation with Excellent Heat Dissipation. Compos. Part A Appl. Sci. Manuf..

[32] Bayan S., Bhattacharya D., Mitra R.K., Ray S.K., Ray S.K. (2020). Self-Powered Flexible Photodetectors Based on Ag Nanoparticle-Loaded g-C3N4nanosheets and PVDF Hybrids: Role of Plasmonic and Piezoelectric Effects. Nanotechnology.

[33] Kar E., Bose N., Dutta B., Mukherjee N., Mukherjee S. (2017). Poly(Vinylidene Fluoride)/Submicron Graphite Platelet Composite_ A Smart, Lightweight Flexible Material with Significantly Enhanced β Polymorphism, Dielectric and Microwave Shielding Properties. Eur. Polym. J..

[34] Agbabiaka O.G., Adegun M.H., Chan K.Y., Zhang H., Shen X., Kim J.K. (2022). BN-PVDF/RGO-PVDF Laminate Nanocomposites for Energy Storage Applications. Nanomaterials.

[35] Chen Y., Guerin S., Yuan H., O’Donnell J., Xue B., Cazade P.A., Haq E.U., Shimon L.J.W., Rencus-Lazar S., Tofail S.A.M. (2022). Guest Molecule-Mediated Energy Harvesting in a Conformationally Sensitive Peptide-Metal Organic Framework. J. Am. Chem. Soc..

[36] Chu R., Weng L., Guan L., Liu J., Zhang X., Wu Z. (2023). Preparation and Properties Comparison of ZIF-67/PVDF and SiCNWs/PVDF Composites for Energy Storage. J. Mater. Sci. Mater. Electron..

[37] Yan Z., Yang Y., Cai X. (2020). Preparation of a Ferroelectric Composite Film Metal–Organic Framework/PVDF. J. Polym. Res..

[38] Sinha C., Mandal D., Roy K., Jana S., Ghosh S.K., Mahanty B., Mallick Z., Sarkar S. (2020). Three-Dimensional MOF-Assisted Self-Polarized Ferroelectret: An Effective Autopowered Remote Healthcare Monitoring Approach. Langmuir.

[39] Roy K., Jana S., Mallick Z., Ghosh S.K., Dutta B., Sarkar S., Sinha C., Mandal D. (2021). Two-Dimensional MOF Modulated Fiber Nanogenerator for Effective Acoustoelectric Conversion and Human Motion Detection. Langmuir.

[40] Guan L., Weng L., Chen N., Kannan H., Li Q., Zhang X., Wu Z., Ma Y., Sahu A. (2021). Bimetallic Organic Framework NiFeMOF Driven by Tiny Ag Particles for PVDF Dielectric Composites. Compos. Part A Appl. Sci. Manuf..

[41] Asadi K., van der Veen M.A. (2016). Ferroelectricity in Metal–Organic Frameworks: Characterization and Mechanisms. Eur. J. Inorg. Chem..

[42] Sasmal A., Sen S., Arockiarajan A. (2022). Strategies Involved in Enhancing the Capacitive Energy Storage Characteristics of Poly(Vinylidene Fluoride) Based Flexible Composites. ChemistrySelect.

[43] Bi M., Hao Y., Zhang J., Lei M., Bi K. (2017). Particle Size Effect of BaTiO_3_ Nanofillers on the Energy Storage Performance of Polymer Nanocomposites. Nanoscale.

[44] Sahu M., Vivekananthan V., Hajra S., Abisegapriyan K.S., Maria Joseph Raj N.P., Kim S.J. (2020). Synergetic Enhancement of Energy Harvesting Performance in Triboelectric Nanogenerator Using Ferroelectric Polarization for Self-Powered IR Signaling and Body Activity Monitoring. J. Mater. Chem. A.

[45] Sun F., Wang G., Ding Y., Wang C., Yuan B., Lin Y. (2018). NiFe-Based Metal–Organic Framework Nanosheets Directly Supported on Nickel Foam Acting as Robust Electrodes for Electrochemical Oxygen Evolution Reaction. Adv. Energy Mater..

[46] Mesbah A., Rabu P., Sibille R., Mazet T., Malaman B., Franc M., Lamour I.J., Cnrs U.M.R., De Lorraine U., Botanique J. (2014). From Hydrated Ni_3_(OH)_2_(C_8_H_4_O_4_)_2_(H_2_O)_4_ to Anhydrous Ni_2_(OH)_2_(C_8_H_4_O_4_): Impact of Structural Transformations on Magnetic Properties. Inorg. Chem..

[47] Martins P., Lopes A.C., Lanceros-Mendez S. (2014). Electroactive Phases of Poly(Vinylidene Fluoride): Determination, Processing and Applications. Prog. Polym. Sci..

[48] Cai X., Lei T., Sun D., Lin L. (2017). A Critical Analysis of the α, β and γ Phases in Poly(Vinylidene Fluoride) Using FTIR. RSC Adv..

[49] Mondal S., Paul T., Maiti S., Das B.K., Chattopadhyay K.K. (2020). Human Motion Interactive Mechanical Energy Harvester Based on All Inorganic Perovskite-PVDF. Nano Energy.

[50] Maity S., Sasmal A., Sen S. (2023). Barium Titanate Based Paraelectric Material Incorporated Poly(Vinylidene Fluoride) for Biomechanical Energy Harvesting and Self-Powered Mechanosensing. Mater. Sci. Semicond. Process..

[51] Chinya I., Pal A., Sen S. (2017). Polyglycolated Zinc Ferrite Incorporated Poly(Vinylidene Fluoride)(PVDF) Composites with Enhanced Piezoelectric Response. J. Alloys Compd..

[52] Srivastava A., Jana K.K., Maiti P., Kumar D., Parkash O. (2015). Poly(Vinylidene Fluoride)/CaCu_3_Ti_4_O_12_ and La Doped CaCu_3_Ti_4_O_12_ Composites with Improved Dielectric and Mechanical Properties. Mater. Res. Bull..

[53] Cao Q., Zhu W., Chen W., Chen X., Yang R., Yang S., Zhang H., Gui X., Chen J. (2022). Nonsolid TiOxNanoparticles/PVDF Nanocomposite for Improved Energy Storage Performance. ACS Appl. Mater. Interfaces.

[54] Xie Y., Wang J., Yu Y., Jiang W., Zhang Z. (2018). Enhancing Breakdown Strength and Energy Storage Performance of PVDF-Based Nanocomposites by Adding Exfoliated Boron Nitride. Appl. Surf. Sci..

[55] Wang H.Q., Wang J.W., Wang X.Z., Gao X.H., Zhuang G.C., Yang J.B., Ren H. (2022). Dielectric Properties and Energy Storage Performance of PVDF-Based Composites with MoS_2_@MXene Nanofiller. Chem. Eng. J..

[56] Wu L., Wu K., Liu D., Huang R., Huo J., Chen F., Fu Q. (2018). Largely Enhanced Energy Storage Density of Poly(Vinylidene Fluoride) Nanocomposites Based on Surface Hydroxylation of Boron Nitride Nanosheets. J. Mater. Chem. A.

[57] Pan Z., Ding Q., Yao L., Huang S., Xing S., Liu J., Chen J., Zhai J. (2019). Simultaneously Enhanced Discharge Energy Density and Efficiency in Nanocomposite Film Capacitors Utilizing Two-Dimensional NaNbO_3_@Al_2_O_3_ Platelets. Nanoscale.

[58] Sasmal A., Sen S., Devi P.S. (2020). Frequency Dependent Energy Storage and Dielectric Performance of Ba-Zr Co-Doped BiFeO_3_loaded PVDF Based Mechanical Energy Harvesters: Effect of Corona Poling. Soft Matter.

[59] Sasmal A., Patra A., Arockiarajan A. (2022). Tuning the Space Charge Polarization of PVDF Based Ternary Composite for Piezo-Tribo Hybrid Energy Harvesting. Appl. Phys. Lett..

[60] Lee K.Y., Kim D., Lee J.H., Kim T.Y., Gupta M.K., Kim S.W. (2014). Unidirectional High-Power Generation via Stress-Induced Dipole Alignment from ZnSnO_3_ Nanocubes/Polymer Hybrid Piezoelectric Nanogenerator. Adv. Funct. Mater..

[61] Muhammad H., Yaseen A., Park S. (2023). Enhanced Power Generation by Piezoelectric P(VDF-TrFE)/RGO Nanocomposite Thin Film. Nanomaterials.

[62] Mondal A., Faraz M., Khare N. (2022). Magnetically Tunable Enhanced Performance of CoFe_2_O_4_-PVDF Nanocomposite Film-Based Piezoelectric Nanogenerator. Appl. Phys. Lett..

[63] Sasmal A., Patra A., Maity S., Pratihar S., Sen S. (2022). Multiferroic BiFeO_3_-Based Hydrophobic Polymer Composites for Polarization Rationalization-Induced Piezo-Tribo Hybrid Energy Harvesting and Versatile Self-Powered Mechanosensing. Sustain. Energy Fuels.

[64] Paria S., Si S.K., Karan S.K., Das A.K., Maitra A., Bera R., Halder L., Bera A., De A., Khatua B.B. (2019). A Strategy to Develop Highly Efficient TENGs through the Dielectric Constant, Internal Resistance Optimization, and Surface Modification. J. Mater. Chem. A.

[65] Zou H., Zhang Y., Guo L., Wang P., He X., Dai G., Zheng H., Chen C., Wang A.C., Xu C. (2019). Quantifying the Triboelectric Series. Nat. Commun..

[66] Zhou Q., Takita R., Ikuno T. (2023). Improving the Performance of a Triboelectric Nanogenerator by Using an Asymmetric TiO_2_/PDMS Composite Layer. Nanomaterials.

[67] Chung M.H., Kim H.J., Yoo S., Jeong H., Yoo K.H. (2022). Enhancement of Triboelectricity Based on Fully Organic Composite Films with a Conducting Polymer. RSC Adv..

[68] Singh H.H., Khare N. (2018). Flexible ZnO-PVDF/PTFE Based Piezo-Tribo Hybrid Nanogenerator. Nano Energy.

[69] Kang X., Pan C., Chen Y., Pu X., Kang X., Chen Y., Pu X., Pan C., Pu X. (2020). Boosting Performances of Triboelectric Nanogenerators by Optimizing Dielectric Properties and Thickness of Electrification Layer. RSC Adv..

[70] Singh H.H., Kumar D., Khare N. (2021). Tuning the Performance of Ferroelectric Polymer-Based Triboelectric Nanogenerator. Appl. Phys. Lett..

[71] Paranjape M.V., Graham S.A., Patnam H., Manchi P., Yu J.S. (2022). Dopamine Treated SnO_2_/PVDF Composite Films for Hybrid Mechanical Energy Harvester. Compos. Sci. Technol..

[72] He W., Qian Y., Lee B.S., Zhang F., Rasheed A., Jung J.E., Kang D.J. (2018). Ultrahigh Output Piezoelectric and Triboelectric Hybrid Nanogenerators Based on ZnO Nanoflakes/Polydimethylsiloxane Composite Films. ACS Appl. Mater. Interfaces.

[73] Roy K., Ghosh S.K., Sultana A., Garain S., Xie M., Bowen C.R., Henkel K., Schmeiβer D., Mandal D. (2019). A Self-Powered Wearable Pressure Sensor and Pyroelectric Breathing Sensor Based on GO Interfaced PVDF Nanofibers. ACS Appl. Nano Mater..

